# A randomized, placebo-controlled repeat-dose thorough QT study of inhaled loxapine in healthy volunteers

**DOI:** 10.5414/CP202457

**Published:** 2015-10-07

**Authors:** James V. Cassella, Daniel A. Spyker, Paul P. Yeung

**Affiliations:** 1 Alexza Pharmaceuticals, Inc., Mountain View, CA, and; 2 Teva Pharmaceuticals, Frazer, PA, USA

**Keywords:** inhalation, loxapine, pharmacodynamics, repeat dose, thorough QT/QTc

## Abstract

Objective: This randomized, double-blind, active- and placebo-controlled, crossover, thorough QT study assessed the effect of two inhaled loxapine doses on cardiac repolarization as measured by corrected QT (QTc) interval in healthy subjects (ClinicalTrials.gov NCT01854710). Methods: Subjects received two doses of inhaled loxapine (10 mg) 2 hours apart + oral placebo, two doses of inhaled placebo + oral placebo, or two doses of inhaled placebo + oral moxifloxacin (400 mg; positive control), with ≥ 3 days washout between treatments. Two-sided 90% confidence intervals (CIs) were calculated around least-squares mean predose placebo-subtracted individually corrected QT durations (ΔΔQTcIs) at 12 time points throughout 24 hours after dosing. A ΔΔQTcI 95% upper CI exceeding 10 msec was the threshold indicating QTc prolongation (primary endpoint). Secondary endpoints included Fridericia- and Bazett-corrected QT duration and QTcI outliers. Pharmacokinetics and adverse events (AEs) were also assessed. Results: Of 60 subjects enrolled (mean age, 33.8 years; 52% male), 44 completed the study. Post loxapine dosing, no ΔΔQTcI 95% upper CI exceeded 10 msec; the largest was 6.31 msec 5 minutes post dose 2. Methodology was validated by ΔΔQTcI 95% lower CIs exceeding 5 msec at 9 of 12 time points after moxifloxacin dosing. Loxapine plasma concentrations increased rapidly (mean C_max_, 177 ng/mL; median t_max_ 2 minutes after dose 2, 2.03 hours after dose 1). There were no deaths, serious AEs, or AEs leading to discontinuation, and one severe AE. Conclusions: Primary and secondary endpoints indicated two therapeutic doses of inhaled loxapine did not cause threshold QTc prolongation in this study.

## Introduction 

Loxapine is an antipsychotic that has been used as an oral schizophrenia treatment for ~ 40 years [1]. It exhibits both typical and atypical antipsychotic properties, displaying clinically relevant inhibition of both the dopamine D_2_/D_3_ and the serotonin 5-HT_2A_ receptors [2]. A new formulation of loxapine (inhaled loxapine aerosol for deep lung absorption) has been approved in the United States (USA) and European Union (EU) for the rapid treatment of agitation in adults with schizophrenia and bipolar disorder. In three double-blind randomized placebo-controlled trials in patients with schizophrenia [3, 4] or bipolar disorder [5] and with clinically relevant agitation, inhaled loxapine 10 mg significantly improved agitation vs. placebo and was well tolerated. 

Delivery of the new formulation utilizes the Staccato^®^ hand-held device, which creates thermally generated loxapine aerosol particles that are then inhaled. Approximately 90% of this loxapine dose is absorbed into the systemic circulation, with plasma concentrations peaking 2 minutes after dosing [6] and clinical effects seen by 10 minutes after dosing [5]. 

Despite the oral formulation being available for 40 years, the effect of loxapine on cardiac repolarization has only recently been studied. Antipsychotic use in general has been implicated in cardiac safety concerns, associated in particular with torsades de pointes (TdP), a ventricular arrhythmia that can degenerate into fibrillation and sudden cardiac death [7]. Prolongation of the corrected QT (QTc) interval is a risk factor and surrogate marker for potential TdP. QTc prolongation may be congenital or acquired, and risk factors include electrolyte imbalances, systemic and cardiac diseases, and the use of certain medications. The relationships between antipsychotic use, QTc prolongation, TdP, and sudden cardiac death are not well defined and vary considerably among the antipsychotic agents [8]. The potency and therefore expected plasma levels of an antipsychotic agent are likely to have an impact. In-vitro studies have suggested that the extent of human ether-a-go-go-related gene (hERG) channel inhibition is a key factor affecting QTc prolongation [9]. Furthermore, it has been demonstrated in-vitro for a number of antipsychotics that a < 10-fold selectivity for dopamine D_2_ or serotonin 2A receptors relative to the hERG channel was associated with QT interval prolongation [10]. 

Although loxapine has not been identified specifically as a risk factor for TdP, investigation of its effect on QTc is warranted. In addition, the International Conference on Harmonization (ICH) Guideline E14 requires a thorough QT/QTc study for approval of reformulated drugs [11]. Accordingly, a premarketing thorough QT study (Phase 1, single center, double-blind, double-dummy, active- and placebo-controlled, three-period crossover study) was performed in which 47 healthy subjects received a single dose of inhaled loxapine 10 mg [12]. This study demonstrated that administration of a single dose of inhaled loxapine 10 mg does not prolong QTc interval to the level of concern. However, in the EU, administration of a second dose of inhaled loxapine 10 mg 2 hours after the first is permitted, and the potential effects of a repeat dose on cardiac repolarization are unknown. 

This randomized, double-blind, active- and placebo-controlled crossover study was designed to assess the maximum effect of inhaled loxapine on cardiac repolarization (QTc interval duration) after two doses administered 2 hours apart compared with placebo in healthy subjects. The relationships between QTc and the concentrations of loxapine and 8-hydroxy-loxapine (8-OH-loxapine) were also assessed. 

## Methods 

### Study design 

This study was conducted at a single clinical research unit in the Netherlands (Pharmaceutical Research Associates International, Inc., Zuidlaren) with ethical approval from an Independent Ethics Committee (Medisch Ethische Toetsings Commissie, Stichting Beoordeling Ethiek Biomedisch Onderzoek, Assen, The Netherlands) in accordance with the Declaration of Helsinki, EU regulations, and the ICH E6 Good Clinical Practice: Consolidated Guidance. Subjects provided written informed consent. It was a phase 1, single center, randomized, double-blind, double-dummy, active- and placebo-controlled, three-period, two-dose, crossover QTc and pharmacokinetics (PK) study in healthy male and female subjects (ClinicalTrials.gov identifier, NCT01854710), designed in accordance with the ICH E14 criteria for a thorough QT study. 

Eligible participants were male and female volunteers aged between 18 and 65 years inclusive, with body mass index ≥ 18 and ≤ 32 kg/m^2^, who were in general good health according to investigator opinion following a detailed medical history and physical examination. Principal exclusion criteria included regular consumption of ≥ 5 cups of coffee per day; a positive alcohol, cotinine, or urine drug test; smoking in the last 30 days; a current history of, or use of medications for, asthma, chronic obstructive lung disease, any other lung disease associated with bronchospasm, or any acute respiratory signs/symptoms (e.g., wheezing); a history within the past 2 years of drug or alcohol dependence or abuse; a history of bronchospasm, allergy, or intolerance following loxapine or amoxapine; acute illness or medication (other than ongoing oral contraception, or ibuprofen or acetaminophen for pain) within the last 5 days; a history of unstable angina, syncope, coronary artery disease, myocardial infarction, congestive heart failure, transient ischemic attack, or neurological disorders; incompatibility with loxapine or the inhalation device in the investigator’s opinion; hypotension (systolic blood pressure (BP), ≤ 90 mmHg; diastolic BP, ≤ 50 mmHg) or hypertension (systolic BP, ≥ 140 mmHg; diastolic BP, ≥ 90 mmHg); or an electrocardiogram (ECG) abnormality. A normal ECG was defined as QTc ≤ 450 milliseconds (msec) for males and ≤ 470 msec for females. Subjects had to have consistent sinus rhythm, heart rate (HR) ≤ 99 and ≥ 40 beats per minute, PR interval between 120 and 230 msec, QRS interval ≤ 120 msec, no other conduction abnormalities, and QT intervals that could be consistently analyzed. 

### Dosing 

The new loxapine formulation (Adasuve^®^) uses the Staccato inhalation delivery system to rapidly deliver loxapine systemically. It is a single-use hand-held product with a medical-grade plastic housing that channels airflow during inhalation. A breath sensor detects inhalation, which activates the controlled gasless redox reaction inside the heat source, causing a rapid rise in temperature. The excipient-free loxapine coating the external surface of the heat source vaporizes in < 1 second, and cools and condenses, while still within the device, into particles 1.0 – 3.5 µm in diameter that are carried deep into the lung with a single inhalation [13]. The inhaled placebo was an identical Staccato device without the loxapine coating on the external surface of the heat source. The following three treatments were administered: 2 × inhaled loxapine 10 mg 2 hours apart plus oral placebo (treatment A); 2 × inhaled Staccato placebo 2 hours apart plus oral placebo (treatment B); 2 × inhaled Staccato placebo 2 hours apart plus oral moxifloxacin 400 mg (treatment C). Subjects were randomized to receive the three treatments according to 1 of 6 sequences (e.g., ABC, ACB) using a computer-generated randomization sequence, with each treatment separated by a washout period of ≥ 3 days. 

### Endpoints 

The primary endpoint tested if the maximum effect of two doses of inhaled loxapine 10 mg on predose subtracted individually corrected QT (QTcI) duration compared with placebo (ΔΔQTcI) upper 95% confidence bound was ≥ 10 msec at any of 12 postdose time points. The individual correction was based on the regression of QTc vs. RR interval during the baseline day preceding the first dose of study medication. Secondary endpoints included: maximum effect of inhaled loxapine on period-specific predose subtracted Fridericia-corrected QTc (QTcF) and Bazett-corrected QTc (QTcB) intervals; numbers and percentages of subjects with QTcI > 450 msec, QTcI > 480 msec, and QTcI > 500 msec; the maximum observed changes from baseline in QTcI; and the numbers and percentages of subjects with QTcI increases > 30 msec and > 60 msec. 

### Assessments 

QT assessment was derived from continuous 12-lead Holter recordings performed for each treatment period from ~45 minutes predose until 22 hours after administration of dose 2. During ECG sampling times subjects were supine with limited activity, and no other study activities were performed. ECG data were analyzed independently by Cardiocore Lab, LLC, Bethesda, MD, USA, where the cardiologists were blinded to period, sequence, and treatment. A single cardiologist read all of the ECGs from an assigned subject. Separate 12-lead ECGs were taken and monitored at the clinical research unit for safety. 

Concentrations of plasma loxapine and its major metabolite 8-OH-loxapine were measured using an established validated method. 

### Safety analysis 

Safety measures included adverse events (AEs), pre- and post-treatment ECGs (HR, PR interval, and QRS outliers), routine clinical laboratory testing (blood chemistry, hematology, and urinalysis), vital sign measurements, and physical examinations. AE recording continued to 30 days after the last dose of study drug, with longer follow-up if necessary. 

### Statistics 

A sample size of 42 subjects was calculated to provide 90% power to reject the primary hypothesis that the true difference from placebo is no more than 3 msec [14]. Enrollment of at least 48 subjects was planned to allow for dropouts. 

The safety population comprised all randomized subjects who received at least one dose of study drug, while all subjects who received study drug and provided a PK sample were included in the PK population. All subjects who received at least placebo and one dose of inhaled loxapine and who had at least one set of time-matched placebo and treatment ECG assessments were included in the QT analyses. All subjects who completed the inhaled loxapine treatment period were included in the concentration vs. QT analysis. 

ECG QT and RR measurements were conducted in the composite 12-lead superimposed global view using high-resolution manual on-screen calipers in the semiautomatic mode with fiducial annotations over-read and adjusted as necessary in the treatment-blinded environment. Each QTc was corrected according to QTcI, QTcB, and QTcF correction formulae. The predose baseline was the average of nine ECGs immediately before dosing. The average of triplicate ECGs from predetermined time points post treatment and at equivalent times on the baseline day served as each subject’s time-controlled QT values. 

A repeated-measures, mixed-effects, linear model was used to calculate least-squares means corrected for sequence, period, and predose baseline, together with corresponding two-sided 90% confidence intervals (CIs) for ΔΔQTcI (the primary endpoint) at each time point. If no upper CIs exceeded 10 msec, then there was no threshold pharmacologic effect of loxapine on QT interval. Secondary endpoints (changes from period-specific predose baseline in QTcF, QTcB, and HR) were analyzed using the same statistical methods. Secondary analysis also included categorical analyses of QTcI, QTcF, and QTcB. 

Assay sensitivity was demonstrated using moxifloxacin 400  mg, a dose known to prolong QTcI. Using the same model as the primary endpoint, two-sided 90% CIs were constructed on the predose-corrected mean QTcI difference between moxifloxacin and the corresponding time-matched, predose-corrected placebo. Assay sensitivity was confirmed if the lower bound exceeded 5 msec at ≥ 1 time point between 1.5 and 3.0 hours post dose. 

Plasma concentrations of loxapine and 8-OH-loxapine were analyzed using noncompartmental methods with WinNonlin^®^ (Version 5.2; extravascular model). Geometric mean and percent coefficient of variation (CV%) were used to characterize peak plasma concentration (C_max_); C_max_ after each dose (C_max1_, C_max2_); apparent terminal half-life of loxapine and 8-OH-loxapine; and apparent clearance (CL/F) of loxapine; and the median was used to describe time to reach C_max_ (t_max_). The relationship between ΔΔQTcI (dependent variable) and loxapine concentration (independent variable) was determined using a linear mixed-effects model. 

All safety analyses, summary tables, and individual subject data listings were carried out using SAS software, version 9.2. 

## Results 

### Subject description and disposition 

**Figure 1.**
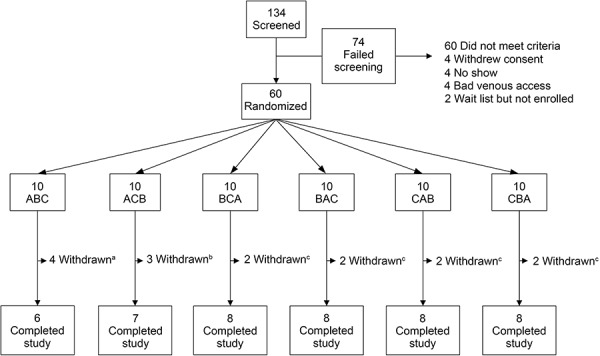
Subject disposition (safety population). Treatment: A = inhaled loxapine 2 × 10 mg; B = placebo; C = oral moxifloxacin 400 mg. ^a^Two subjects withdrawn owing to procedural error by the CRU and two subjects withdrawn owing to subject request after treatment A. ^b^Two subjects withdrawn owing to procedural error by the CRU and one subject withdrawn owing to subject request after treatment A. ^c^Two subjects withdrawn owing to procedural error by the CRU. CRU = clinical research unit.

Of the 134 screened subjects, 60 were enrolled and received at least one dose of study drug (safety population) (Figure 1). There were 45 subjects who received loxapine and contributed at least one loxapine concentration (PK population), and 44 subjects had at least one set of time-matched placebo and inhaled loxapine ECGs (QT population). 

The mean ± standard deviation age of the enrolled subjects was 33.8 ± 14.9 years; 51.7% were male, 88.3% were white, and 73.3% had never smoked (Table 1). 

### Primary QTcI endpoint and assay sensitivity 

**Figure 2.**
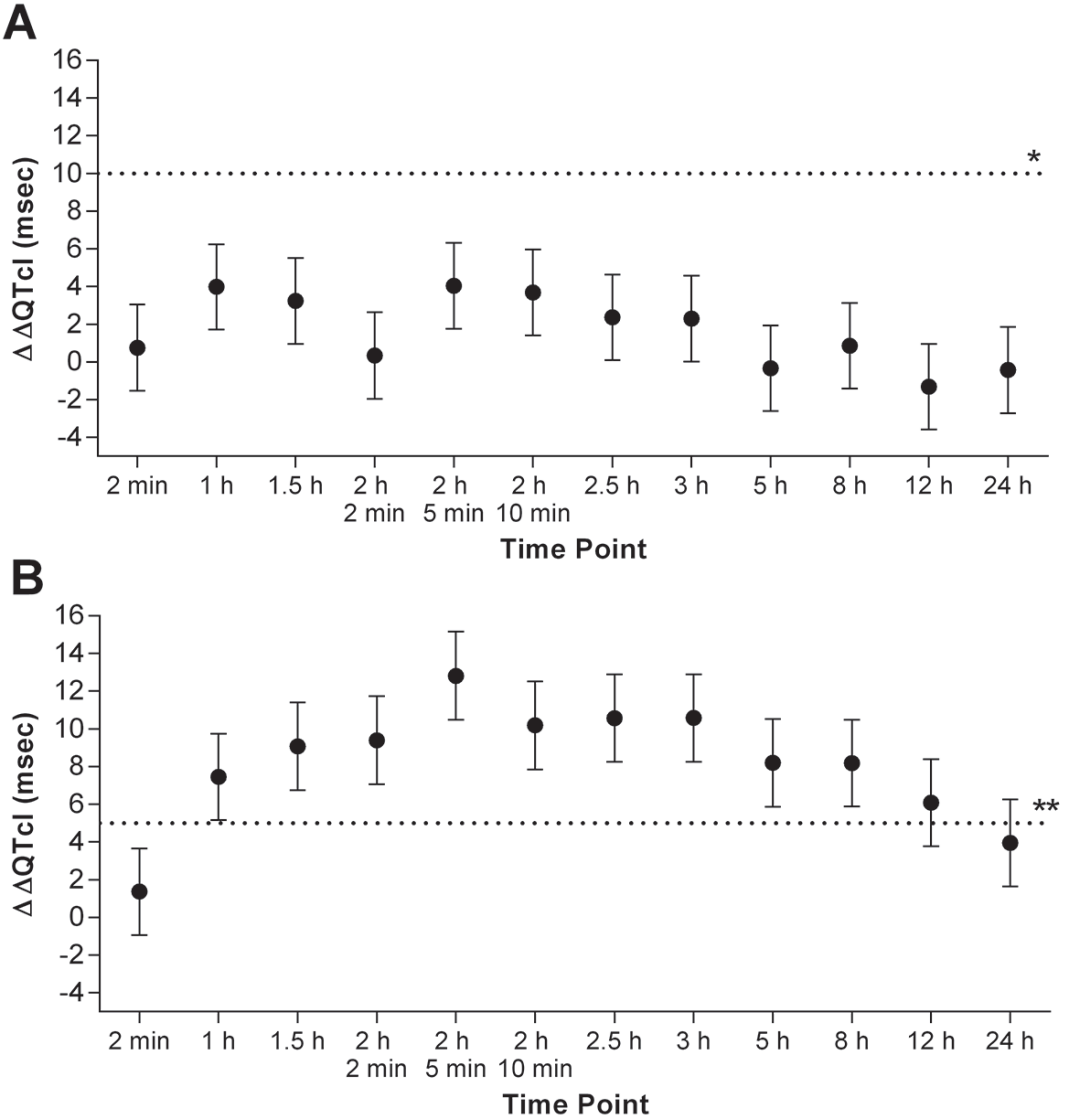
Least-squares mean ΔΔQTcI (predose subtracted individually corrected QT duration compared with placebo) and two-sided 90% CIs, primary analysis model (QT population). A: Inhaled loxapine. *Reference line at 10 msec shows the threshold of regulatory concern for the upper bound of the 95% CI on the mean effect of the primary outcome measure (QTcI). B: Oral moxifloxacin. **Reference line shows the threshold for concluding suitable assay sensitivity if any moxifloxacin QTcI lower 95% CI exceeds 5 msec. CI = confidence interval.

The one-sided 95% upper confidence bound for each ΔΔQTcI after two doses of inhaled loxapine 10 mg did not exceed 10 msec at any of the 12 postdose time points (Figure 2A). The largest upper confidence bound was 6.31 msec at 5 minutes post dose 2. The effect of this treatment on QT/QTc prolongation is less than the specified threshold pharmacologic effect on cardiac repolarization and represents a negative thorough QT/QTc study, consistent with ICH E14 criteria [11]. The one-sided 95% lower confidence bound of the ΔΔQTcI exceeded 5 msec at 9 of the 12 time points post moxifloxacin (Figure 2B), thus demonstrating assay sensitivity. 

In general gender had no effect on QTcI. There was a statistically significant sex-by-treatment group interaction (p = 0.035) at 24 hours where females and males showed a –3.3 and +2.1 msec difference from placebo, respectively. 

### Secondary QT endpoints 

An identical statistical analysis of QTcF and QTcB showed that the largest one-sided 95% upper confidence bounds were 6.54 msec at 1 hour post dose for ΔΔQTcF and 7.67 msec at 24 hours post dose for ΔΔQTcB. Both values were < 10 msec, supporting the primary QT analysis findings. There were no significant differences from placebo in QTcI outliers after inhaled loxapine dosing. 

### PK results 

Loxapine was not detected in any predose plasma sample. After administration, loxapine was absorbed rapidly into the plasma, with a median (minimum, maximum) t_max_ of 0.03 (0.03, 1.03) hours after dose 1 and 0.03 (0.03, 0.12) hours after dose 2, with plasma concentrations decreasing rapidly thereafter. Mean (CV%) loxapine C_max_ was 108 (34.4%) ng/mL after the first dose, 176 (29.5%) ng/mL after the second dose, and 177 (26.7%) ng/mL overall. Plasma concentrations of the major metabolite, 8-OH-loxapine, were C_max1_, 5.08 (36.0%) ng/mL and C_max2_, 9.20 (33.4%) ng/mL. The mean loxapine overall C_max_ value was ~ 19-fold higher than that observed for 8-OH-loxapine. Mean (CV%) half-life was 8.96 (18.9%) hours for loxapine and 18.6 (28.1%) hours for 8-OH-loxapine, and mean (CV%) apparent clearance for loxapine was 55.7 (22.9%) L/h. 

### Plasma drug levels and QTcI 

**Figure 3.**
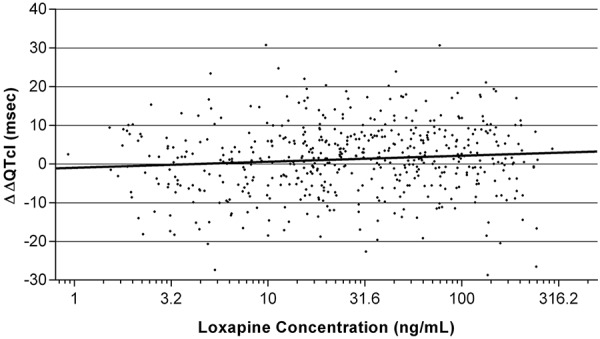
Predose subtracted individually corrected QT duration compared with placebo (ΔΔQTcI, msec) vs. loxapine concentrations (ng/mL) and fitted linear regression (ΔΔQTcI = –1.1 + 1.64 (log (loxapine)); p = 0.013).

**Figure 4.**
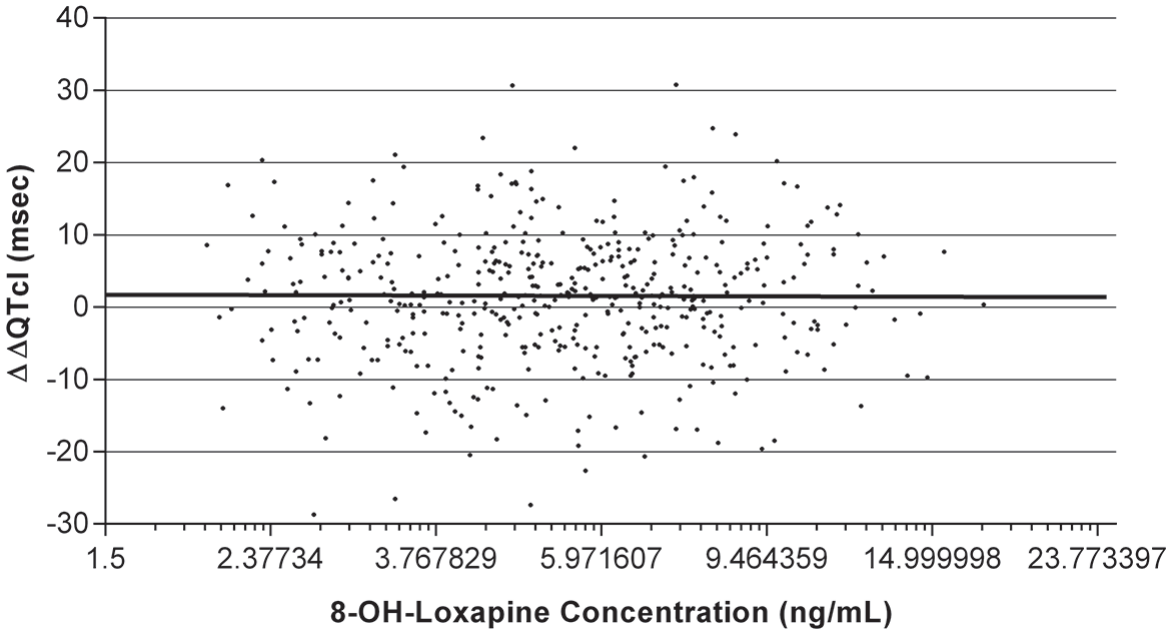
Predose subtracted individually corrected QT duration compared with placebo (ΔΔQTcI, msec) vs. 8-OH-loxapine concentrations (ng/mL) and fitted linear regression (ΔΔQTcI = 1.7 – 0.561 (log (loxapine)); p = not significant). 8-OH-loxapine = 8-hydroxy loxapine.

The regression of ΔΔQTcI and log loxapine concentrations was linear, with a positive slope (p = 0.013) (Figure 3). However, the highest value for the 95% CI upper bound at the highest observed concentration of loxapine (293 ng/mL) was 4.6 msec, consistent with the negative thorough QT/QTc study result. There was no statistically significant relationship (zero slope regression) between ΔΔQTcI and log 8-OH-loxapine concentration (Figure 4). 

### Safety 

There were no deaths, serious AEs, or AEs leading to discontinuation. One subject experienced one severe AE after the second inhaled loxapine dose (oculogyric crisis), which resolved after 9 hours. All other AEs were mild or moderate. Most (80.8%) subjects reported an AE after receiving inhaled loxapine, compared with 49.0% after receiving placebo and 34.7% after receiving moxifloxacin. Treatment-related AEs were more frequent after inhaled loxapine dosing (73.1%) compared with placebo (30.6%) and moxifloxacin (18.4%). The most frequent (at least five subjects) treatment-related AEs following loxapine administration were sedation, fatigue, dizziness, dysgeusia, and somnolence (Table 2). There were no respiratory AEs (coughs, dyspnea, wheezing, or bronchospasm). Hypotension was reported in two subjects after receiving inhaled loxapine; both events resolved within 20 minutes. Tachycardia was reported in 2 subjects after the second dose of inhaled loxapine; both events were mild and resolved after 2 and 9 hours. 

There were no significant effects on the numbers of PR and QRS outliers from the 12-lead Holter core lab analyses. There were no clinically significant mean changes in HR, respiratory rate, temperature, or BP. No clinically significant laboratory abnormalities were reported. 

## Discussion 

This randomized, double-blind, double-dummy, active- and placebo-controlled, three-period crossover thorough QT/QTc study was designed in accordance with ICH E14 guidelines to assess the potential for two doses of inhaled loxapine 10 mg to delay cardiac repolarization by measuring QTc duration. The findings from this study demonstrate that, in healthy subjects, two doses of inhaled loxapine 10 mg administered 2 hours apart did not cause threshold QTc prolongation according to ICH criteria, and were well tolerated. 

A sufficient number of subjects completed the study to achieve the objectives. The validity of the sample size selected and the methodology used was demonstrated by the effects of the oral moxifloxacin 400 mg dose (positive control). The effects of moxifloxacin on QTc interval are well characterized and were detected as expected in this study [15]. Assay sensitivity was demonstrated by moxifloxacin prolonging QTcI. 

This study met the primary QTcI endpoint and is therefore a negative thorough QT/QTc study according to the ICH E14 criteria, meaning that two doses of inhaled loxapine administered 2 hours apart did not cause a threshold prolongation in QTc. The secondary endpoints (QTcF, QTcB, and categorical changes in QTcI) all supported the primary endpoint analysis, and there was no overall difference between male and female response (no sex-by-treatment interaction). 

Although loxapine was administered in this study at twice the total dose used in the previous single 10 mg dose thorough QT study [12], these ΔΔQTcI findings are broadly comparable, confirming the negative thorough QT/QTc conclusions of both studies. 

There was a small but statistically significant positive relationship between QTcI and log loxapine concentration, which differed slightly from the single-dose thorough QT study, where the relationship was nonlinear and downwardly parabolic [12]. However, when the present results were examined for the most extreme (minimum and maximum) loxapine concentrations, the absence of expected clinical effect was confirmed. There was no relationship between QTcI and metabolites of loxapine in either this two-dose (8-OH-loxapine) or in the single-dose (7-OH-loxapine) thorough QT study. The PK results also confirmed previous findings, showing that inhaled loxapine rapidly enters the systemic circulation (median t_max_ of 2 minutes after each dose) and is rapidly distributed and cleared thereafter [6]. Two thorough QT/QTc studies with similar results, as discussed here, means that the effect of inhaled loxapine on cardiac repolarization is well studied compared with other antipsychotics, which typically have only one, or zero, thorough QTc studies [8]. 

Overall, two doses of inhaled loxapine 10 mg administered 2 hours apart were well tolerated in healthy subjects. The most frequent treatment-related AEs were consistent with those previously reported for loxapine (sedation, fatigue, dizziness, and somnolence) and for inhaled drugs (dysgeusia) [12]. 

The lack of a threshold effect on QTc interval may be due to loxapine’s low level of hERG blocking at therapeutic doses compared with other antipsychotic agents. An in-vitro study showed that loxapine dose-dependently blocked the hERG channel with a half-maximal inhibitory concentration of 1,800 nM (unpublished data, submitted to regulatory authorities [16]). In comparison, the hERG half-maximal inhibitory concentration values of other antipsychotic agents include: sertindole, 3 nM; droperidol, 32 nM; risperidone, 167 nM; ziprasidone, 169 nM; thioridazine, 191 nM; perphenazine, 1003 nM; chlorpromazine, 1,561 nM; quetiapine, 5,765 nM; and olanzapine, 6,013 nM [10, 17]. Cardiac safety concerns linked to QTc prolongation and TdP have led to various withdrawals, warnings, and restricted use of sertindole, droperidol, and thioridazine in the EU and US [18]. The loxapine dose used in this study represents the maximum recommended exposure and is the regimen approved in the EU. There have been some reports of cardiac AEs occurring after oral loxapine overdose [19, 20, 21], a situation unlikely to arise with a dose-limiting inhaled device administered intermittently solely in health care facilities. When considering medication to treat agitation in emergency settings, the risk of QTc prolongation should be considered, especially because additional TdP risk factors such as concomitant drug use and underlying disease may be present [22]. In this respect, inhaled loxapine may be preferential to other drugs commonly used, such as haloperidol, which has a Food and Drug Administration warning for QTc prolongation and TdP, especially at high doses [23, 24]. 

The limitations of this study include the use of healthy subjects, which precludes observation of drug-induced QTc prolongation in a population with additional factors predisposing to TdP (including hypokalemia and underlying heart conditions). Subjects with respiratory disease were excluded, consistent with the contraindications on the product label. However, despite some concerns regarding potential bronchospasm induction by inhaled loxapine [25], no airway-related AEs were observed in this study. 

In conclusion, this thorough QT study, which was validated using moxifloxacin as a positive control, showed that in healthy subjects, two doses of inhaled loxapine 10 mg administered 2 hours apart did not cause threshold QTc prolongation and were well tolerated. Additionally, the QTc changes seen here were broadly comparable to the previous study, which tested a single dose of inhaled loxapine 10 mg, contributing to the conclusion that inhaled loxapine is not associated with cardiac repolarization liability. These two thorough QT studies make inhaled loxapine one of the most thoroughly studied antipsychotics with regard to QTc prolongation. 

## Conflict of interest 

JVC and DAS were employees of Alexza Pharmaceuticals during execution of this study, are currently paid consultants of Alexza Pharmaceuticals, and have received stock and stock options. PPY is a full-time employee of Teva Pharmaceuticals and receives stock options from Teva Pharmaceuticals. This study was funded by Alexza Pharmaceuticals. 

## Acknowledgments 

Medical writing support was provided by Christine Tomlins, PhD of Excel Scientific Solutions, Horsham, UK, and was funded by Teva Pharmaceuticals. 

**Table 1.** Description of study participants (safety population).

Subject characteristic	Placebo (n = 49)	Inhaled loxapine 2 × 10 mg (n = 52)	Oral moxifloxacin 400 mg (n = 49)	Overall (N = 60)
Sex, n (%)				
Female	24 (49.0)	25 (48.1)	24 (49.0)	29 (48.3)
Male	25 (51.0)	27 (51.9)	25 (51.0)	31 (51.7)
Age (years)				
Mean (SD)	34.0 (14.6)	34.1 (15.1)	33.9 (15.0)	33.8 (14.9)
Race, n (%)				
White	43 (87.8)	46 (88.5)	44 (89.8)	53 (88.3)
Black	1 (2.0)	1 (1.9)	0	2 (3.3)
Native American	2 (4.1)	2 (3.8)	2 (4.1)	2 (3.3)
Other	3 (6.1)	3 (5.8)	3 (6.1)	3 (5.0)
Smoking history, n (%)				
Never smoked	38 (77.6)	39 (75.0)	37 (75.5)	44 (73.3)
Ex-smoker	11 (22.4)	13 (25.0)	12 (24.5)	16 (26.7)

SD = standard deviation.

**Table 2.** Treatment-related AEs experienced in at least two subjects after any treatment (safety population).

System organ class adverse event, n (%)	Placebo^a^ (n = 49)	Inhaled loxapine 2 × 10 mg (n = 52)	Oral moxifloxacin 400 mg (n = 49)
Subjects with any treatment-related AE	15 (30.6)	38 (73.1)	9 (18.4)
Cardiac disorders			
Tachycardia	0	2 (3.8)	0
Eye disorders			
Asthenopia	0	2 (3.8)	0
Gastrointestinal disorders			
Dry mouth	0	4 (7.7)	0
Dysgeusia	3 (6.1)	8 (15.4)	2 (4.1)
Nausea	0	3 (5.8)	0
Paresthesia oral	0	2 (3.8)	0
General disorders and administration site conditions			
Fatigue	2 (4.1)	13 (25.0)	2 (4.1)
Nervous system disorders			
Disturbance in attention	0	2 (3.8)	0
Dizziness	4 (8.2)	12 (23.1)	0
Headache	5 (10.2)	3 (5.8)	2 (4.1)
Presyncope	0	2 (3.8)	0
Restlessness	0	2 (3.8)	0
Sedation	4 (8.2)	14 (26.9)	2 (4.1)
Somnolence	1 (2.0)	5 (9.6)	1 (2.0)
Vascular disorders			
Hypotension	0	2 (3.8)	0

AE = adverse event. ^a^Placebo includes exposure for the placebo treatment period and exposure to oral placebo before inhaled loxapine exposure.

## References

[1] Heel RC Brogden RN Speight TM Avery GS Loxapine: a review of its pharmacological properties and therapeutic efficacy as an antipsychotic agent. Drugs. 1978; 15: 198–217. 25167 10.2165/00003495-197815030-00002

[2] Kapur S Zipursky R Remington G Jones C McKay G Houle S PET evidence that loxapine is an equipotent blocker of 5-HT2 and D2 receptors: implications for the therapeutics of schizophrenia. Am J Psychiatry. 1997; 154: 1525–1529. 9356559 10.1176/ajp.154.11.1525

[3] Allen MH Feifel D Lesem MD Zimbroff DL Ross R Munzar P Spyker DA Cassella JV Efficacy and safety of loxapine for inhalation in the treatment of agitation in patients with schizophrenia: a randomized, double-blind, placebo-controlled trial. J Clin Psychiatry. 2011; 72: 1313–1321. 21294997 10.4088/JCP.10m06011yel

[4] Lesem MD Tran-Johnson TK Riesenberg RA Feifel D Allen MH Fishman R Spyker DA Kehne JH Cassella JV Rapid acute treatment of agitation in individuals with schizophrenia: multicentre, randomised, placebo-controlled study of inhaled loxapine. Br J Psychiatry. 2011; 198: 51–58. 21200077 10.1192/bjp.bp.110.081513

[5] Kwentus J Riesenberg RA Marandi M Manning RA Allen MH Fishman RS Spyker DA Kehne JH Cassella JV Rapid acute treatment of agitation in patients with bipolar I disorder: a multicenter, randomized, placebo-controlled clinical trial with inhaled loxapine. Bipolar Disord. 2012; 14: 31–40. 22329470 10.1111/j.1399-5618.2011.00975.x

[6] Spyker DA Munzar P Cassella JV Pharmacokinetics of loxapine following inhalation of a thermally generated aerosol in healthy volunteers. J Clin Pharmacol. 2010; 50: 169–179. 19915181 10.1177/0091270009347866

[7] Glassman AH Bigger JT Antipsychotic drugs: prolonged QTc interval, torsade de pointes, and sudden death. Am J Psychiatry. 2001; 158: 1774–1782. 11691681 10.1176/appi.ajp.158.11.1774

[8] Hasnain M Vieweg WV QTc interval prolongation and torsade de pointes associated with second-generation antipsychotics and antidepressants: a comprehensive review. CNS Drugs. 2014; 28: 887–920. 25168784 10.1007/s40263-014-0196-9

[9] Crumb WJ Ekins S Sarazan RD Wikel JH Wrighton SA Carlson C Beasley CM Effects of antipsychotic drugs on I(to), I (Na), I (sus), I (K1), and hERG: QT prolongation, structure activity relationship, and network analysis. Pharm Res. 2006; 23: 1133–1143. 16715368 10.1007/s11095-006-0070-7

[10] Kongsamut S Kang J Chen XL Roehr J Rampe D A comparison of the receptor binding and HERG channel affinities for a series of antipsychotic drugs. Eur J Pharmacol. 2002; 450: 37–41. 12176106 10.1016/s0014-2999(02)02074-5

[11] International Conference on Harmonisation; guidance on E14 Clinical Evaluation of QT/QTc Interval Prolongation and Proarrhythmic Potential for Non-Antiarrhythmic Drugs; availability. Notice. Fed Regist. 2005; 70: 61134–61135. 16237860

[12] Spyker DA Voloshko P Heyman ER Cassella JV Loxapine delivered as a thermally generated aerosol does not prolong QTc in a thorough QT/QTc study in healthy subjects. J Clin Pharmacol. 2014; 54: 665–674. 24375070 10.1002/jcph.257

[13] Dinh K Myers DJ Glazer M Shmidt T Devereaux C Simis K Noymer PD He M Choosakul C Chen Q Cassella JV In vitro aerosol characterization of Staccato( ) Loxapine. Int J Pharm. 2011; 403: 101–108. 20971174 10.1016/j.ijpharm.2010.10.030

[14] Zhang J Machado SG Statistical issues including design and sample size calculation in thorough QT/QTc studies. J Biopharm Stat. 2008; 18: 451–467. 18470755 10.1080/10543400802020938

[15] Shah RR Morganroth J Kleiman RB ICH E14 Q&A(R2) document: commentary on the further updated recommendations on thorough QT studies. Br J Clin Pharmacol. 2015; 79: 456–464. 25060671 10.1111/bcp.12477 PMC4345956

[16] http://www.ema.europa.eu/docs/en_GB/document_library/EPAR_-_Public_assessment_report/human/002400/WC500139407.pdf and http://www.accessdata.fda.gov/drugsatfda_docs/nda/2012/022549Orig1s000PharmR.pdf accessed Oct 2014..

[17] Drolet B Zhang S Deschênes D Rail J Nadeau S Zhou Z January CT Turgeon J Droperidol lengthens cardiac repolarization due to block of the rapid component of the delayed rectifier potassium current. J Cardiovasc Electrophysiol. 1999; 10: 1597–1604. 10636190 10.1111/j.1540-8167.1999.tb00224.x

[18] Pae CU Sertindole: dilemmas for its use in clinical practice. Expert Opin Drug Saf. 2013; 12: 321–326. 23432404 10.1517/14740338.2013.773971

[19] Peterson CD Seizures induced by acute loxapine overdose. Am J Psychiatry. 1981; 138: 1089–1091. 7258388 10.1176/ajp.138.8.1089

[20] Roberge RJ Martin TG Mixed fluoxetine/loxapine overdose and atrial flutter. Ann Emerg Med. 1994; 23: 586–590. 8135440 10.1016/s0196-0644(94)70083-4

[21] Mazzola CD Miron S Jenkins AJ Loxapine intoxication: case report and literature review. J Anal Toxicol. 2000; 24: 638–641. 11043672 10.1093/jat/24.7.638

[22] Baker SN Management of acute agitation in the emergency department. Adv Emerg Nurs J. 2012; 34: 306–318. 23111305 10.1097/TME.0b013e31826f12d6

[23] Sharma ND Rosman HS Padhi ID Tisdale JE Torsades de Pointes associated with intravenous haloperidol in critically ill patients. Am J Cardiol. 1998; 81: 238–240. 9591913 10.1016/s0002-9149(97)00888-6

[24] http://www.fda.gov/Drugs/DrugSafety/PostmarketDrugSafetyInformationforPatientsandProviders/DrugSafetyInformationforHeathcareProfessionals/ucm085203.htm accessed Sep 2014..

[25] Gross N Greos LS Meltzer EO Spangenthal S Fishman RS Spyker DA Cassella JV Safety and tolerability of inhaled loxapine in subjects with asthma and chronic obstructive pulmonary disease – two randomized controlled trials. J Aerosol Med Pulm Drug Deliv. 2014; 27: 478–487. 24745666 10.1089/jamp.2013.1114 PMC4273199

